# The Association Between Endurance Training and Heart Rate Variability: The Confounding Role of Heart Rate

**DOI:** 10.3389/fphys.2018.00756

**Published:** 2018-06-19

**Authors:** David Herzig, Babken Asatryan, Nicolas Brugger, Prisca Eser, Matthias Wilhelm

**Affiliations:** University Clinic for Cardiology, Inselspital, Bern University Hospital, University of Bern, Bern, Switzerland

**Keywords:** heart rate variability, heart rate, autonomic nervous system, endurance training, bradycardia

## Abstract

Heart rate variability (HRV) is a widely used marker of cardiac autonomic nervous activity (CANA). Changes in HRV with exercise training have often been interpreted as increases in vagal activity. HRV is strongly associated with heart rate, which in turn, is associated with heart size. There is strong evidence from basic studies that lower heart rate in response to exercise training is caused by morphological and electrical remodeling of the heart. In a cross-sectional study in participants of a 10 mile race, we investigated the influence of endurance exercise on HRV parameters independently of heart size and heart rate. One-hundred-and-seventy-two runners (52 females and 120 males) ranging from novice runners with a first participation to an endurance event to highly trained runners, with up to 15 h of training per week, were included in the analysis. R-R intervals were recorded by electrocardiography over 24 h. Left ventricular end diastolic volume indexed to body surface area (LVEDVI) was assessed by transthoracic echocardiography and peak oxygen consumption (VO_2_peak) by cardiopulmonary exercise testing. Exercise was quantified by VO_2_peak, training volume, and race performance. HRV was determined during deep sleep. HRV markers of vagal activity were moderately associated with exercise variables (standardized β = 0.28–0.40, all *p* < 0.01). These associations disappeared when controlling for heart rate and LVEDVI. Due to the intrinsic association between heart rate and HRV, conclusions based on HRV parameters do not necessarily reflect differences in CANA. Based on current evidence, we discourage the use of HRV as a marker of CANA when measuring the effect of chronic exercise.

## Introduction

Heart rate variability (HRV) is widely used as a marker of the cardiac autonomic nervous system activity (CANA). Especially, beat-to-beat variability of heart rate seems to be modulated by vagal activity (Akselrod et al., 1985; Pomeranz et al., 1985; Pagani et al., 1986). These findings come from acute experiments conducted to assess how heart rate is controlled by the CANA using pharmacological blockades (Pagani et al., 1986), direct neural stimulation (Chess et al., 1975), or selective denervation in animal studies (Chiou and Zipes, 1998). However, it should not be assumed that underlying mechanisms for chronic change in HRV are identical to those of acute change (Mourot et al., 2004; Valenzano et al., 2016). Nevertheless, many studies have reported an increase in HRV parameters quantifying beat-to-beat variability and therefore suggested a causative role of CANA (al-Ani et al., 1996; Levy et al., 1998; Iwasaki et al., 2003; Madden et al., 2006).

An often neglected, but possibly important, role may be held by heart rate (Sacha and Pluta, 2008; Monfredi et al., 2014; Kazmi et al., 2016). While a change in CANA will elicit changes in both heart rate and HRV, heart rate is also affected by other factors, namely intrinsic heart rate. Also, the variability of heart rate has been shown to be dependent on the diastolic depolarization rate (Wilders and Jongsma, 1993; Rocchetti et al., 2000). Thus, beat-to-beat variability is tightly linked to heart rate, which itself is highly dependent on the electrical properties of the sinoatrial node. In cases of non-autonomic changes in heart rate, this could lead to misinterpretation of study results.

At the moment there is a heated debate on the possible origins of exercise-induced bradycardia (Boyett et al., 2017), wherein the effect of exercise training on vagal activity has been questioned and the leading role of morphological and electrical remodeling in this bradycardia has been postulated. There is strong evidence of exercise-induced electrical remodeling of the sinus node (D’Souza et al., 2014). Conversely, the evidence for a role of the CANA in exercise-induced bradycardia is based on indirect markers of CANA. The findings of changes in HRV with exercise training have been used to ascertain the role of the CANA (Coote and White, 2015; Billman, 2017). However, differences in HRV may, due to its relationship with heart rate, not reflect any actual differences in CANA.

The evidence of non-autonomic change in heart rate following exercise training and the direct influence of this heart rate change on HRV parameters questions the validity of HRV as marker of the CANA to assess effects of exercise training. Therefore, in a population of moderately to highly trained runners, we aimed to investigate if exercise training (quantified by several variables of performance and training volume) influences HRV parameters independently of its influence on heart rate. The amount of variance of HRV parameters shared by exercise and heart rate would be congruent with the argument of electrical and possibly morphological remodeling of the heart induced by exercise training.

## Materials and Methods

### Participants and Protocol

Amateur runners were recruited during a 10 mile race for two studies on exercise and atrial remodeling as previously described (Wilhelm et al., 2011; Brugger et al., 2014). Athletes with a history of cardiovascular disease, regular medication intake (incl. non-steroidal anti-inflammatory drugs), or arterial hypertension, defined as a blood pressure (BP) ≥ 140/90 mmHg at the initial visit (Mancia et al., 2013) were excluded. A total of 227 runners were included in the study. Twenty-four runners were excluded (14 had undiagnosed arterial hypertension, 8 could not participate in the race because of muscular problems, 1 had mitral valve prolapse, and 1 had hypercholesterolemia). All athletes provided written informed consent. The study protocol was approved by the local ethics committee.

Height and weight were measured based on standard procedures. Body surface area (BSA) was calculated using the DuBois formula (DuBois and DuBois, 1916). BP measurements were taken, after resting 5 min in supine position, three times at the right arm with an oscillometric device (Dinamap XL; Criticon Inc., Tampa, FL, United States). The first measurement was discarded and the mean of the two last measurements were used for data analysis (Mancia et al., 2013).

### Training Variables

Average weekly endurance training hours and the variable “training years” (calculated from 18 years onwards) were ascertained by a training questionnaire. Cumulative training was calculated using the following formula: average endurance training hours per week ^∗^ 52 ^∗^ training years (Wilhelm et al., 2011; Brugger et al., 2014).

### Race Performance

Official 10 mile race time was taken from the race results.

### Cardiopulmonary Exercise Test

Cardiopulmonary exercises tests were performed only in a subset of runners. Tests were performed on a treadmill (Woodway PPS 70 Med, Waukesha, WI, United States). We used an incremental protocol starting at 7.2 km^∗^h-1, with the speed increasing 0.2 km^∗^h-1 every 20 s until volitional exhaustion. The respiratory parameters were recorded breath-by-breath with an open spirometry system (CS 200, Schiller-Reomed AG, Dietikon, Switzerland). Data was further process in MATLAB (2014a, The Mathworks Inc., Natick, MA, United States) with a custom-made procedure. Peak oxygen consumption (VO_2_peak) was calculated as the highest 30-s-average value of VO_2_ and expressed relative to body weight (ml^∗^kg^-1∗^min^-1^). Maximal speed was defined as the speed of the last completed step of the protocol.

### Transthoracic Echocardiography (TTE)

Standard two-dimensional TTE was performed on a Phillips iE33 System (X5-1 transducer, Phillips Medical Systems, Zurich, Switzerland). Left ventricular end-diastolic volume (LVEDV) was calculated using the biplane method of disks summation technique and indexed for BSA (LVEDI). Volume measurements were based on tracings of the blood–tissue interface in the apical four- and two-chamber views. At the mitral valve level, the contour was closed by connecting the two opposite sections of the mitral ring with a straight line (Lang et al., 2015).

### Resting Heart Rate and HRV Assessment

A three channel ECG was recorded at a sampling rate of 1000 Hz during 24 h (Lifecard CF, Spacelabs Healthcare, Nuremberg, Germany). Interbeat intervals (R-R intervals) of each recording were exported to MATLAB for further processing. We have developed an algorithm to identify segments of deep sleep characterized by regular breathing and stationary R-R intervals (Herzig et al., 2016). Pearson correlation coefficients of consecutive R-R intervals (rRR) were calculated of 5-min windows moved in steps of 20 s over the whole night. The linear trend of rRR over the first 4 h was removed and we identified the first period where rRR was 0.1 below the mean rRR (which was 0 due to the detrending) for at least 10 min. A 5-min segment was placed in the middle of the identified period. Of the selected segments, time and frequency analysis of the R-R intervals was performed.

### HRV Analysis

The following time domain parameters were calculated: heart rate (beats^∗^min^-1^), the square root of the mean squared differences of adjacent R-R intervals (RMSSD, ms) and the standard deviation of all R-R intervals (SDNN, ms). For spectral analysis, R-R intervals were interpolated using a cubic spline interpolation and resampled at 4 Hz. We applied an advanced smoothness prior approach for detrending of R-R intervals with a smoothing parameter of λ = 500. We used an artifact correction algorithm that eliminated R-R intervals in case of deviations of 30% or more of adjacent R-R intervals and replaced them using a cubic-spline interpolation. Power spectral density was then calculated using Fast Fourier Transformation. Frequency domain parameters were: total power (TP, ms^2^, 0–0.4 Hz), low-frequency power (LF, ms^2^, 0.04–0.15 Hz), high-frequency power (HF, ms^2^, 0.15–0.4 Hz) and the LF/HF power ratio (Camm, 1996). Due to the very high correlation between RMSSD and HF power (in our data, *r* = 0.96), only results for RMSSD will be shown in the results and discussed. However, the results for HF power are similar to the results for RMSSD and our findings are also applicable for HF power.

### Statistical Analysis

Statistical analysis was performed using the software R (Version 3.4.0, R Core Team, 2017). Median values of the measured parameters and interquartile range where calculated for both sexes. Linear models were performed to assess the relationship between exercise and HRV parameters, with separate models for each individual HRV parameter as dependent parameter. Simple regression models were performed with the variables of interest entered together with sex as a covariate. Further, multiple regression were performed with all variables of interest (heart rate, LVEDVI, sex, and a single exercise variable) entered together as independent parameters. These exercise variables were parameters of training history (weekly training and cumulative training) and performance (VO_2_peak and 10 mile race time). Further, linear models were performed for heart rate as dependent variable and heart size, sex and a single exercise parameter as independent variables. For the multiple regression models semi-partial R^2^ were calculated. The semi-partial R^2^ corresponds to the variance uniquely described by this parameter. 90% CI for the semi-partial R^2^ were computed using a non-central distribution as described by Smithson (2001) (we use 90% CI because R^2^ is always positive and these intervals correspond to a two-sided test at a 95% confidence level for variables that can be positive or negative). Diagnostic plots of all performed models were visually inspected with regard to satisfaction of underlying statistical assumptions. Data were log transformed if necessary. Collinearity was assessed by the variation inflation factor (all vif < 2.2). A *p*-value of less than 0.05 was considered statistically significant.

## Results

### Participants

A total of 203 subjects completed the study. Data of 31 runners was discarded due to insufficient quality of the data in the segment selected for HRV analysis (less than 90% of valid data or non-stationary signal). Thus, data of 172 normotensive runners (52 females and 120 males) was included in the analyses. Cardiopulmonary exercise tests were performed only in a subset of runners, therefore VO_2_peak data were available of 96 runners (51 females and 45 males). Median weekly endurance training hours was 4.0 h ranging from 0 to 15 h, and cumulative lifetime training hours ranged from 0 to 17576 h. Baseline characteristics for both sexes are summarized in **Table 1**.

**Table 1:** Baseline characteristics.

		Female	IQR	Male	IQR
*Baseline*					
	*n*	52		120	
	Age (years)	42	[37; 48]	42	[36; 47]
	Weight (kg)	56.1	[53.0; 60.8]	74.3	[69.0; 81.0]
	Height (cm)	168	[162; 171]	178	[174; 183]
	BMI (kg^∗^cm^-2^)	20.2	[19.6;21.1]	22.5	[21.6; 23.9]
	Systolic BP	110	[100; 118]	124	[115; 129]
	Diastolic BP	70	[65; 70]	76	[73; 80]
*HRV*					
	*n*	52		120	
	Heart rate (bpm)	54.7	[49.5; 61.7]	52.1	[46.8; 59.4]
	LF (ms^2^)	278	[132; 534]	324	[216; 563]
	HF (ms^2^)	441	[260; 754]	408	[190; 711]
	Total power (ms^2^)	797	[421; 1307]	744	[774; 1195]
	LF/HF	0.61	[0.36; 0.85]	0.88	[0.46; 1.64]
	RMSSD (ms)	46.4	[30.6; 62.3]	40.6	[28.8; 56.2]
	SDNN (ms)	40.8	[30.5; 51.6]	38.7	[32.7; 51.6]
*CPET Test*					
	*n*	51		45	
	VO_2_peak (ml^∗^mn^-1∗^kg^-1^)	50.4	[45.5; 55.1]	55.5	[48.6; 59.6]
	VO_2_peak absolute (ml^∗^mn^-1^)	2837	[2619; 3032]	4154	[3715; 4423]
	Maximal speed (km^∗^h^-1^)	15.6	[14.7; 16.8]	18.3	[16.6; 20.0]
*Performance*					
	*n*	50		115	
	10 mile race time (min)	79	[71; 98]	69	[62; 74]
*Training*					
	*n*	52		120	
	Weekly endurance training (hours)	5	[4; 6]	4	[3; 5]
	Cumulative training (hours)	3120	[2145; 5772]	3328	[2080; 5460]
	Endurance training duration (years)	11	[9; 16]	13	[10; 21]
*Echocardiography*					
	*n*	52		120	
	LVEDV (ml)	81.0	[70.8; 91.3]	114.1	[96.0; 124.5]
	LVEDVI (ml^∗^m^-2^)	49.6	[45.2; 55.8]	58.0	[50.9; 65.2]

*Data are presented as median and interquartile range (IQR); BMI, Body mass index; BP, Blood Pressure; LF, low-frequency power; HF, high-frequency power; LF/HF, ratio of low-frequency power to high-frequency power; RMSSD, square root of the mean squared differences of adjacent R-R intervals; SDNN, standard deviation of all R-R intervals; LVEDV, left ventricular end diastolic volume; LVEDVI, left ventricular end diastolic volume indexed to body surface area.*

### Heart Rate and HRV

Heart rate variability parameters as well as 10 mile race time were non-normally distributed. For consistency reasons, all parameters in **Table 1** are reported with their medians and interquartile range. For the linear regression models, HRV parameters and 10 mile race time were log transformed using the natural logarithm.

### Relationship Between Exercise and HRV and Between Exercise and Heart Rate

Both training variables and both measured performance variables were significantly related to RMSSD (all *p* < 0.01) with standardized betas (β) ranging from a magnitude of 0.28 to 0.40 in the models with sex as a covariate. The highest explained variance (R^2^) was observed for the model with VO_2_peak (*R*^2^ = 14.9%). The other models explained around 10% (*R*^2^ ranging from 8.8 to 12.7%) of the variance in RMSSD. Similar relationships were observed between the exercise variables and heart rate, albeit with lower β (ranging from 0.24 to 0.29). Weak associations between training variables (weekly endurance training and lifetime training hours) and LF/HF ratio were observed (β = -0.15 and β = -0.19, respectively, both *p* < 0.05) while no significant effects of VO_2_peak and 10 mile race time on LF/HF ratio were observed. The model outputs for the independent variable VO_2_peak and weekly endurance training including standardized beta-coefficients, *p*-values and 95% confidence intervals are listed in **Table 2**.

**Table 2:** Linear regression models for HRV parameters and Heart Rate.

		*Models for log(RMSSD)*			*Models for log(LF/HF)*			*Models for Heart Rate*	

**Predictor**	***beta***		***r***	***beta***		***r***	***beta***		***r***
		**95% CI**			**95% CI**			**95% CI**	
		**[LL, UL]**			**[LL, UL]**			**[LL, UL]**	
**Model 1**	*R*^2^ = 0.465^∗∗^ 95% CI[0.29,0.56]			*R*^2^= 0.161^∗∗^ 95% CI[0.02,0.27]			*R*^2^= 0.239^∗∗^ 95% CI[0.09,0.36]		
LVEDVI	0.04	[-0.18, 0.25]	0.38^∗∗^	0.04	[-0.23, 0.31]	-0.04	-0.52^∗∗^	[-0.76, -0.29]	-0.47^∗∗^
Heart rate	-0.57^∗∗^	[-0.75, -0.39]	-0.64^∗∗^	0.19	[-0.03, 0.41]	0.21^∗^			
Sex	-0.18^∗^	[-0.34, -0.02]	-0.14	0.35^∗∗^	[0.14, 0.55]	0.33^∗∗^	0.14	[-0.05, 0.33]	0.03
VO_2_peak	0.19	[-0.01, 0.39]	0.31^∗∗^	-0.11	[-0.36, 0.13]	-0.05	0.04	[-0.19, 0.27]	-0.25^∗^
**Model 2**	*R*^2^= 0.444^∗∗^ 95% CI[0.32,0.52]			*R*^2^= 0.161^∗∗^ 95% CI[0.06,0.25]			*R*^2^= 0.228^∗∗^ 95% CI[0.12,0.32]		
LVEDVI	0.18^∗^	[0.04, 0.32]	0.37^∗∗^	-0.06	[-0.23, 0.12]	-0.07	-0.44^∗∗^	[-0.59, -0.29]	-0.43^∗∗^
Heart rate	-0.50^∗∗^	[-0.63, -0.36]	-0.61^∗∗^	0.23^∗∗^	[0.07, 0.39]	0.27^∗∗^			
Sex	-0.17^∗^	[-0.30, -0.04]	-0.14	0.29^∗∗^	[0.13, 0.44]	0.28^∗∗^	0.13	[-0.01, 0.28]	0.01
Training	0.14	[-0.01, 0.26]	0.35^∗∗^	-0.07	[-0.22, 0.08]	-0.19^∗^	-0.15^∗^	[-0.29, -0.00]	-0.26^∗∗^

*^∗^p < 0.05; ^∗∗^p < 0.01. beta indicates the standardized regression weights; r represents the zero-order correlation. CI, Confidence interval for the beta; LL and UL indicate the lower and upper limits of a confidence interval, respectively. The training variable is the weekly endurance training.*

### Relationship Between Exercise and Heart Size

VO_2_peak was strongly associated with LVEDVI (β = 0.52 ± 0.07, *p* < 0.01) and a slightly weaker association was observed between 10 mile race time and LVEDVI (β = -0.44 ± 0.07, *p* < 0.001). Weekly endurance training and cumulative training were associated with LVEDVI (β = 0.29 ± 0.07, *p* < 0.01 and β = 0.22 ± 0.07, *p* < 0.01, respectively).

### Relationship Between LVEDVI and Heart Rate

A moderate, negative, association between heart rate and LVEDVI (β = -0.44 ± 0.07, *p* < 0.01) was observed.

### Relationship Between Heart Rate and RMSSD and Between Heart Rate and LF/HF

There was a strong negative association between heart rate and RMSSD both in the simple model (with only sex as covariate) and in the complete model were heart rate was entered together with the other variables (β = 0.61 ± 0.06, *p* < 0.01 and β = -0.57 ± 0.06, *p* < 0.01, respectively). A moderate association was observed between heart rate and LF/HF (β = 0.31 ± 0.07, *p* < 0.01).

### Relationship Between Exercise and HRV Independently of Heart Size and Heart Rate

**FIGURE 1:**
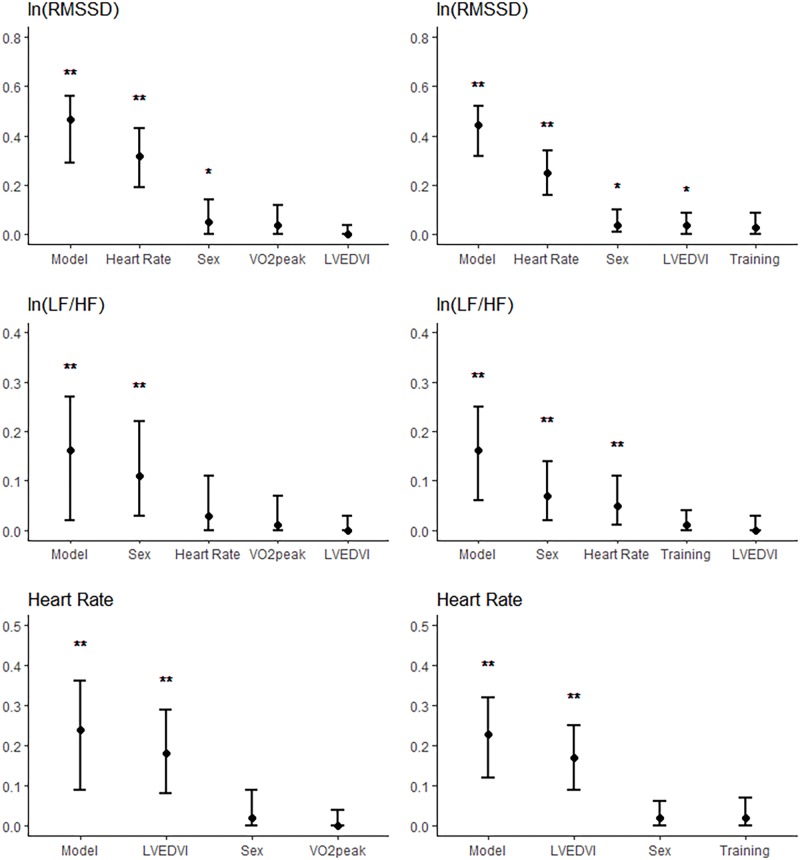
Semi-partial R^2^ of the models for ln(RMSSD) **(Upper)**, ln(LF/HF) **(Middle)**, and heart rate **(Lower)** when entering all predictors into the models. R^2^ of the complete models are also shown (“model”). Figures are shown for the models with VO_2_peak (Left) and weekly endurance training (Right) entered as exercise variables. The whiskers represent the 95% confidence intervals. ^∗^*p* < 0.05; ^∗∗^*p* < 0.01.

Semi-partial R^2^ are displayed in **Figure 1**. In the models for RMSSD, semi-partial R^2^ for all exercise variables were not statistically different from 0. The highest semi-partial R^2^ was observed with VO_2_peak with a semi-partial R^2^ of 4.1%, 90% CI [0.0%, 15.0%]. In the models for RMSSD, the semi-partial R^2^ for LVEDVI ranged between 0 [0.0%, 0.1%] and 3.0% [0.0%, 6.1%]. In the models for LF/HF, none of the exercise variables had a semi-partial R^2^ significantly different from 0. This was also the case for the semi-partial R^2^ of LVEDVI in all performed models. Thus, both the variance of LF/HF explained by any of the exercise variables and the variance explained by LVEDVI were contained within the variance explained by heart rate. In the models for heart rate, semi-partial R^2^ for exercise variables were not significantly different from 0.

## Discussion

The present study tested whether endurance training and/or performance could explain some of the variance of HRV independently of heart rate and heart size in a cohort of amateur runners. Neither training history nor exercise performance were related to RMSSD or to LF/HF ratio independently of heart rate and heart size. Given the evidence of recent studies that exercise training leads to bradycardia by electrical remodeling (D’Souza et al., 2014; Boyett et al., 2017), vagal activity is likely to only play a minor role in explaining exercise-induced bradycardia and high values of RMSSD.

In conditions (such as chronic exercise training), in which electrical remodeling of the sinus node leads to bradycardia, there is an automatic effect on HRV parameters due to the intrinsic link of HRV to HR (Wilders and Jongsma, 1993; Rocchetti et al., 2000; Monfredi et al., 2014). This association, also observed in our data, has been reported to be of biophysical (Monfredi et al., 2014) or mathematical (Sacha and Pluta, 2008) reasons and is independent of CANA.

D’Souza et al. (2014) showed strong evidence for a reduction in heart rate due to structural changes in the sinus node. Their data indicated a training-induced ion channel remodeling through a downregulation of HCN4 (HCN: hyperpolarization-activated cyclic nucleotide-gated channels) channels and of the corresponding ionic current (I_f_), which correlated with the slowing of heart rate. Most studies comparing the association between training and changes in resting heart rate, as well as between changes in intrinsic heart rate, found a greater decrease in intrinsic heart rate than in resting heart rate, which do not support a higher vagal activity due to training (Boyett et al., 2013). However, some studies observed smaller change in intrinsic heart rate compared to resting heart rate (for a summary see Boyett et al., 2013).

While our results cannot rule out a possible role of CANA in the exercise-induced bradycardia, in line with the suggestion by Monfredi et al. (2014), the variance in heart rate is sufficient to explain the observed variance in HRV with exercise training. If we assume that electrical remodeling of the sinus node is largely responsible for the decrease in heart rate with increased training in our study population, then we can explain the most part of the variance in beat-to-beat variability of our population by electrical remodeling without the need of any change in CANA. We conclude that HRV should not be used as an explicit marker of the CANA when assessing chronic effects of exercise where structural changes of the heart are likely to occur.

The absence of association between exercise training and RMSSD when controlling for heart rate cannot *per se* be interpreted as an absence of change in the CANA. An increase in vagal activity would both lower heart rate as well as increase RMSSD and lead to the absence of a significant effect of exercise in a statistical model with heart rate entered as covariable. Consequently, without measurements of intrinsic heart rate, interpretation of differences in HRV are impossible. LF/HF is thought by some authors to reflect sympathovagal balance (Pagani et al., 1986; Malliani et al., 1991) and was reported to be independent of heart rate (Zaza and Lombardi, 2001). A moderate association was observed between heart rate and LF/HF and, again, no independent contribution to the variance in LF/HF could be attributed to exercise when adjusting for heart rate and heart size.

Our results suggest an association between heart rate and heart size independent of exercise training (semi-partial *R*^2^ = 18%, **Figure 1**). In the models with heart size, there was no significant independent contribution of the exercise variables to the variance of heart rate (semi-partial *R*^2^ ≤ 2%, *p* > 0.1). This means that the association between exercise training and heart rate can be explained by differences in heart size. It is well established that highly trained athletes have enlarged hearts and lower heart rates compared to sedentary subjects or to less trained athletes (Fagard et al., 1983; Fagard, 1996; Francavilla et al., 2018). While we cannot be certain about any causal relationship between training, heart size and heart rate, the parallel effect of training on heart size and heart rate suggest an important role of morphological remodeling in the difference in heart rate with training.

A limitation of the present study is the method used for the assessment of training. Self-reported questionnaires are subject to recall bias and have only limited accuracy (Prince et al., 2008). However, a more accurate method would strengthen, but not abolish, an existing association.

## Conclusion

In conclusion, endurance exercise training was associated with HRV as well as with heart size and heart rate. Without definite knowledge of causality, it is impossible to assign a causal relationship of exercise training to CANA. Instead, it may hold true, as suggested by some previous studies, that exercise causes morphological and electrical remodeling of the heart resulting in a lower heart rate and as direct consequence in an increased HRV. HRV parameters cannot be used as explicit markers of the CANA when assessing effects of chronic exercise.

## Author Contributions

DH, PE, and MW designed the study. MW contributed to the data collection. NB performed the echocardiographic measurements. DH performed the data analysis. DH and PE performed the statistical analyses. DH, BA, and PE wrote the manuscript. All authors commented on the manuscript and approved the final version of the manuscript.

## Conflict of Interest Statement

The authors declare that the research was conducted in the absence of any commercial or financial relationships that could be construed as a potential conflict of interest.
